# Pharmacokinetics of daptomycin in patients undergoing low-flow continuous venovenous hemodiafiltration

**DOI:** 10.1186/cc14196

**Published:** 2015-03-16

**Authors:** TI Ide, Y Takesue, K Ikawa, S Nishi

**Affiliations:** 1 Hyogo College of Medicine, Nishinomiya City, Japan; 2 Hiroshima University, Hiroshima, Japan

## Introduction

In daptomycin (DAP), 1,061 mg hour/l of the area under the concentration-time curve (AUC)/MIC was required to obtain clinical success [1], and a trough serum concentration (Cmin) cutoff point of 24.3 g/ml was most significantly associated with CPK elevation [2]. Reportedly, DAP at a recommended dosage of 8 mg/kg is removed in patients undergoing high-flow continuous venovenous hemodiafiltration (CVVHDF) (blood flow and filtration rates were 150 ± 48 and 2 l/hour). In Japan, CVVHDF is preferentially performed with lower flow rates. Investigating effects of flow rate on DAP removal during continuous renal replacement therapy is essential to adjust therapeutic dosages. We aimed to investigate the pharmacokinetics of DAP in CVVHDF patients in this setting.

## Methods

DAP (6 mg/kg) was administered intravenously every 48 hours to CVVHDF patients in the ICU. Blood and filtrate samples were collected at 0, 1, 1.5, 2, 5, 12, 24, and 48 hours after infusion. All collected samples were analyzed using HPLC according to the method of Tobin et al. [3]. Maximum concentration (Cmax), elimination half-life (t1/2), area AUC, Cmin, volume of distribution (Vd), clearance (CL), fraction unbound, and sieving coefficient (Sc) were evaluated. Patient characteristics and CVVHDF parameters including blood, dialysate, and filtration flow rates were recorded.

## Results

Three patients were included in the study. Mean blood, dialysate, and filtration flow rates were 86.7 ± 11.5 ml/minute, 417 ± 29 ml/hour, and 417 ± 29 ml/hour, respectively, confirming that CVVHDF was performed under low-flow setting. Cmax was 50.1 ± 12.7 mg/l (31.9, 70.5, 49.7 mg/l); t1/2, 35.1 ± 34.8 hours (18.6, 11.5, 70.5 hours); AUC, 889 ± 399 mg hour/l (471, 967, 1,260 mg hour/l); Cmin, 16.0 ± 10.3 mg/l (2.3, 24.7, 14.0 mg/l); Vd, 26.0 ± 20.9 l (23.8, 6.34, 47.9 l); CL, 9.47 ± 4.56 ml/minute (14.7, 6.35, 7.37 ml/minute); and fraction unbound, 5.8% (5.7, 4.1, 7.6%). Sc and CL of dialyzer were 0.08 ± 0.03 (0.11, 0.04, 0.07) and 1.20 ± 0.39 ml/minute (1.70, 0.88, 0.96 ml/minute), respectively.

## Conclusion

DAP (6 mg/kg daptomycin every 48 hours) in patients receiving low-flow CVVHDF resulted in showing variability of AUC and avoiding accumulation. Owing to small case numbers, it needs further study.
